# Association of Albumin and Globulin with Mortality Risk in Incident Peritoneal Dialysis Patients

**DOI:** 10.3390/nu14142850

**Published:** 2022-07-12

**Authors:** Kuan-Ju Lai, Yao-Peng Hsieh, Ping-Fang Chiu, Pei-Ru Lin

**Affiliations:** 1 Department of Internal Medicine, Changhua Christian Hospital, Changhua 50006, Taiwan; 120752@cch.org.tw; 2 Department of Internal Medicine, Division of Nephrology, Changhua Christian Hospital, Changhua 50006, Taiwan; 68505@cch.org.tw; 3 School of Medicine, Kaohsiung Medical University, Kaohsiung 80708, Taiwan; 4 School of Medicine, Chung Shan Medical University, Taichung 40201, Taiwan; 5 College of Life Sciences, National Chung Hsing University, Taichung 402, Taiwan; 6 Department of Recreation and Holistic Wellness, MingDao University, Changhua 52345, Taiwan; 7 Big Data Center, Changhua Christian Hospital, Changhua 50006, Taiwan; 183778@cch.org.tw

**Keywords:** albumin, globulin, peritoneal dialysis (PD), cardiovascular disease, chronic kidney disease, mortality

## Abstract

Background: Nutrition and inflammation have been implicated in predicting mortality in patients on peritoneal dialysis (PD). Serum albumin and globulin can be regarded for the nutritional and inflammatory status. However, there is lack of data to evaluate the synergistic effect of albumin and globulin on mortality prediction. Methods: In 554 patients initiating PD from January 2001 to July 2016, we divided them into four groups by the combination of two categories of low vs. high albumin and low vs. high globulin. The median values for albumin and globulin were chosen to classify them into low or high groups. Their associations with all-cause and cardiovascular (CV) mortality were examined in Cox regression models adjusted for confounding clinical and laboratory data. Results: Patients, 52.91 ± 15.2 years old and 47.8% men, had a median (interquartile range) value of 3.3 (2.9–3.8) g/dL for albumin and 2.8 (2.5–3.2) g/dL for globulin, respectively. Patients with low albumin and high globulin had the highest all-cause mortality and CV mortality, with adjusted hazard ratios of 3.87 (95% CI 1.83–8.20, *p* < 0.001) and 5.65 (95% CI 2.23–14.34, *p* < 0.001), respectively, compared with those with a high albumin and low globulin having the lowest mortality rate. Sensitivity analyses further confirmed this relationship. Conclusions: A patient profile of either low albumin or high globulin is linked to a higher risk for mortality, particularly for a profile of both low albumin and high globulin compared with one without either of them. Further studies are needed to explore the mechanisms underlying this phenomenon and how to improve clinical outcomes in those high-risk patients.

## 1. Introduction

The number of patients with chronic kidney disease (CKD) is on the rise globally because of longevity and the high prevalence of comorbidities. CKD is a tremendous global health burden, and it is also associated with socioeconomic problems. Even the early stages of CKD are associated with cognitive dysfunction and a decreased quality of life [1,2]. CKD is regarded as an independent risk factor and accelerator for cardiovascular (CV) disease, which is also the leading cause of mortality in CKD [3]. The prevalence of CKD is estimated to be between 11% and 13%, most of which are stage 3 [4]. As an irreversible disease process, the number of patients with end-stage renal disease (ESRD) requiring dialysis is expected to double to 5.4 million by 2030, and peritoneal dialysis (PD) is one of the accepted modalities of renal replacement therapy worldwide [5].

Besides traditional risk factors such as hypertension, dyslipidemia and CV disease, newly recognized conditions, especially malnutrition and persistent low-grade inflammation, play a pivotal role in the pathogenesis of high cardiovascular morbidity and mortality in the clinical setting of CKD, particularly ESRD [6]. Protein energy consumption (PEW) is a special metabolic state of CKD/ESRD, which is different from malnutrition [7]. The latter indicates that the nutritional intake is insufficient but with an appropriate metabolic response, and PEW is resistant to nutritional supplementation. From the literature, the clinical outcomes associated with PEW and malnutrition include cardiovascular disease, frailty and mortality [8,9,10]. Inflammation and malnutrition always occur concomitantly and affect each other in an amplified manner, leading to poor clinical outcomes.

Albumin and globulin are the two main components of serum protein. Undoubtedly, albumin is regarded as a nutritional index in clinical evaluations. Globulin is mainly composed of immunoglobulin and acute phase proteins, and a higher globulin level reflects a more severe inflammatory status. Both of them can predict patient outcomes independently [11,12]. It is unknown whether using both parameters simultaneously helps to stratify or improve predictions of patient survival. Hence, this study was carried out to assess the effect of the interaction between albumin and globulin on the risk of overall mortality and CV mortality in patients on PD.

## 2. Materials and Method

### 2.1. Participants and Measurements

We retrospectively conducted a study to investigate whether albumin and globulin have a synergistic effect in predicting the risk of death in peritoneal dialysis patients. Patients who commenced PD between January 2001 and July 2016 at Changhua Christian Hospital were qualified for our study. If participants were over 18 years old and had received peritoneal dialysis for at least three months, they were included in our study. The time at risk of mortality was from the index date, defined as the date of initiating PD, until patient death or the end of the study on 31 July 2017, whichever occurred first.

The baseline data were collected for statistical analyses and included demographics, comorbid conditions, pharmacotherapy, laboratory data and PD-related parameters. The patients’ demographics included gender, age and body mass index, and the comorbidities consisted of diabetes mellitus (DM), hypertension and cardiovascular disease. The medicated use of renin–angiotensin system blockade, angiotensin-converting enzyme (ACE) inhibitors or angiotensin II receptor blockers (ARB) was also recorded. PD-related parameters comprised a normalized protein nitrogen appearance (nPNA), residual kidney function, weekly Kt/V urea and a dialysate-to-plasma creatinine ratio at 4 h (D/P (creatinine) at 4 h). The residual kidney function was estimated by determining the mean of the urea and creatinine clearances from a 24-h urine collection. The blood tests included creatinine, albumin, globulin, glutamic-pyruvic transaminase (GPT), white blood cell (WBC) count, hemoglobin, ferritin, cholesterol, triglyceride, intact parathyroid hormone (PTH), calcium and phosphate.

To assess the impact of the interaction between albumin and globulin on patient survival, the study cohort was classified as four groups by albumin and globulin. The median values of albumin and globulin were selected as the cut-off points to make the number of subjects in the four groups more equal, as follows: group A (*n* = 135), albumin ≥ 3.3 g/dL and globulin < 2.8 g/dL; group B (*n* = 165), albumin ≥ 3.3 g/dL and globulin ≥ 2.8 g/dL; group C (*n* = 122), albumin < 3.3 g/dL and globulin < 2.8 g/dL; and group D (*n* = 132), albumin < 3.3 g/dL and globulin ≥ 2.8 g/dL. The leading cause of patient mortality in our cohort was CV mortality. The primary outcome assessed was all-cause mortality, and the secondary outcome was CV mortality. This study was approved and supervised by the Changhua Christian Hospital Institutional Review Board and conducted according to the ethical standards of the Declaration of Helsinki. A retrospective nonintrusive study of anonymous patients conducted in Taiwan does not require informed consent from each participant.

### 2.2. Statistical Analysis

The median values of albumin and globulin were selected to divide all the study subjects into four subgroups, and all the baseline patients’ characteristics were presented as the mean ± standard deviation (SD) or a percentage number for the numerical and categorical data, respectively. The distribution of the baseline patients’ characteristics into the four groups was compared using the chi-square test or Fisher’s exact test for categorical variables and analysis of variance (ANOVA) or Kruskal–Wallis test for continuous variables, as appropriate.

Comparison of the survival status amongst the four groups was made by computing a Kaplan–Meier survival curve, and the statistical significance was determined by the log–rank test. Cox proportional hazard models were performed to assess the associations between the variables and study outcomes, including all-cause and CV mortality. Multivariate-adjusted Cox regression analyses were then used to determine the independent role of the study covariates in the outcomes of interest. The potential adjusted covariates included all the baseline data listed in Table 1 and were incorporated into the multivariate Cox models if the *p*-value in the univariate model was <0.05. The results of the Cox models were shown as the hazard ratio (HR) with a 95% confidence interval (CI). Predictors of a low albumin and high globulin group were examined, using logistic regression analyses with all the other albumin–globulin profiles as the reference, and the results were presented as the odds ratio (OR) with a 95% confidence interval. To test the robustness of our results, the statistical analyses were repeated after we reclassified the study cohort into four subgroups based on the lower normal limit of albumin (3.5 g/dL) and the median value of globulin. All the statistical analyses were carried out using IBM SPSS Statistics for Windows, Version 22.0 (IBM Corp., Armonk, NY, USA) and R version 4.1.2. The association was considered significant if the two-sided *p*-value was <0.05.

## 3. Results

### 3.1. Patients’ Baseline Characteristics

During the study period between January 2001 and July 2016, a total of 554 patients were enrolled for the final analyses. The baseline clinical characteristics stratified by albumin and globulin are presented in Table 1. The median (IQR) of albumin and globulin was 3.3 (2.9–3.8) g/dL and 2.8 (2.5–3.2) g/dL, respectively. Of them, 47.8% were men, and the mean age of the whole study cohort was 52.91 ± 15.54 years. The patients in the group D were the oldest (58.48 ± 15.2 years). Some (132, 23.8%) patients (group D) initiated PD with low albumin and high globulin and were more likely to be older, with a higher prevalence of DM and cardiovascular disease. On the contrary, patients with high albumin and low globulin (group A) were more likely to be younger, with a lower prevalence of DM and cardiovascular disease.

### 3.2. Association of Albumin and Globulin with All-Cause Mortality

**Figure 1:**
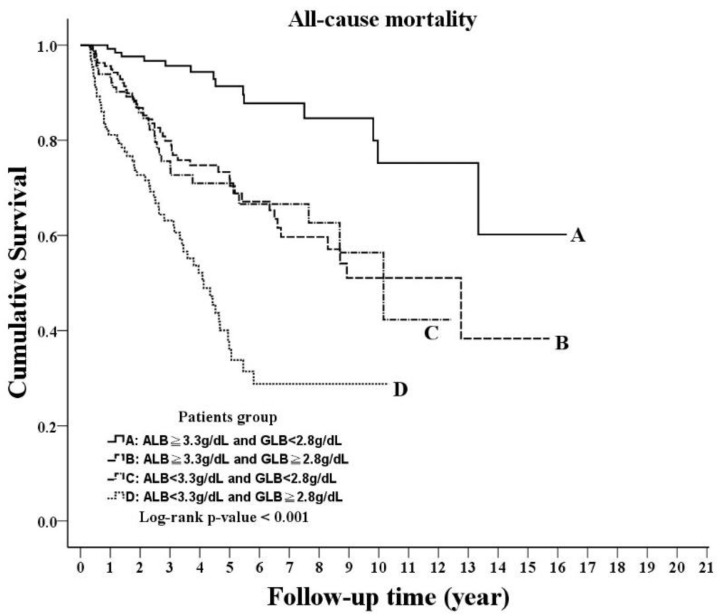
Kaplan–Meier curve of the overall patient survival, according to the groups stratified by albumin and globulin (log–rank test, *p* < 0.001).

During the study period, 151 deaths occurred, yielding a crude all-cause mortality rate of 7.05 per 100 patient-years (95% CI, 5.97–8.27). The crude mortality rates were 2.04 cases per 100 patient-years (95% CI, 1.12–3.43) for group A, 6.80 cases per 100 patient-years (95% CI, 4.96–9.11) for group B, 7.0 cases per 100 patient-years (95% CI, 4.76–9.93) for group C and 17.33 cases per 100 patient-years (95% CI, 13.26–22.26) for group D. The cumulative survival status among the four groups showed significant differences, with a log–rank test *p*-value < 0.001 for the Kaplan–Meier curve (Figure 1). The crude and adjusted HRs for all-cause mortality with reference to group A are shown in Table 2. The HRs for all-cause mortality tended to increase from group A to D (*p* for the trend < 0.001). Compared with group A, the adjusted HRs were 2.74 (95% CI, 1.33–5.65, *p* = 0.006) for group B, 2.22 (95% CI, 1.03–4.82, *p* = 0.043) for group C and 3.87 (95% CI, 1.83–8.20, *p* < 0.001) for group D. The adjusted HR was higher in group D than those in group B and C, indicating a synergistic effect of albumin and globulin in predicting all-cause mortality in PD patients.

### 3.3. Association of Albumin and Globulin with Cardiovascular Mortality

**Figure 2:**
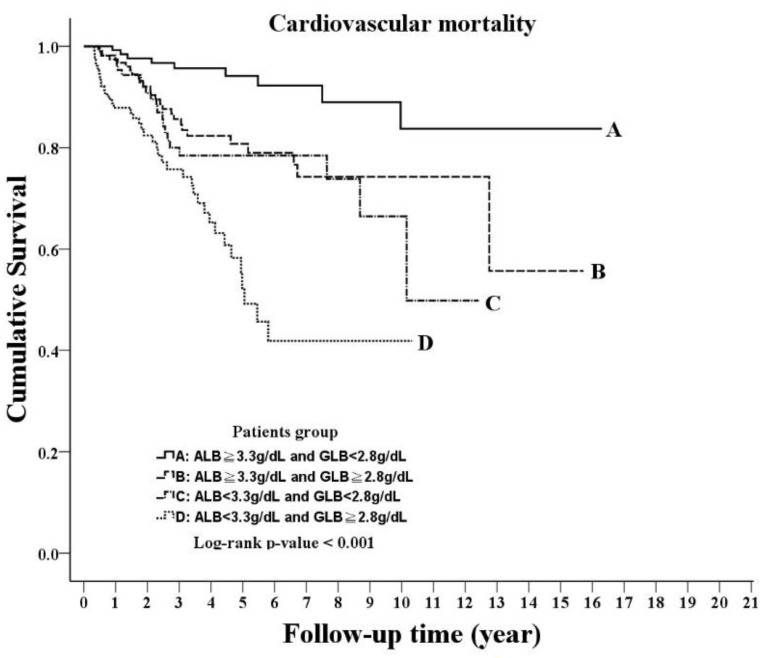
Kaplan–Meier curve of cardiovascular survival according to the groups stratified by albumin and globulin (log–rank test, *p* < 0.001).

Overall, 95 patients died of CVD during the study period, including 9 (6.7%) in group A, 26 (15.8%) in group B, 21 (17.2%) in group C and 39 (29.5%) in group D. A significant difference was noted in the cumulative cardiovascular survival status, as shown in the Kaplan–Meier curve (log–rank test, *p* < 0.001; Figure 2). In both the unadjusted and adjusted Cox models of CV mortality, the HRs showed an increasing trend from group A to group D (both *p* for the trend < 0.001; Table 2). Compared with group A, the adjusted HRs were 2.67 (95% CI, 1.05–6.82, *p* = 0.04) for group B, 2.56 (95% CI, 0.96–6.87, *p* = 0.061) for group C and 5.65 (95% CI, 2.23-14.34, *p* < 0.001) for group D.

### 3.4. Predictors of a Low Albumin and High Globulin Profile

In the adjusted logistic regression models, compared with all the other albumin–globulin profiles, DM and CVD and higher WBC counts were associated with higher adjusted odds of initiating PD with low albumin and high globulin (odds ratio (OR) (95% CI), 2.00 (1.22–3.27); 1.66 (1.01–2.74) and 1.14 (1.04–1.25), respectively; Table 3).

In turn, in both the unadjusted and fully adjusted models, the level of serum creatinine was associated with lower odds of a low albumin and high globulin profile (OR (95% CI), 0.81 (0.75–0.88) and 0.86 (0.79–0.95), respectively).

### 3.5. Sensitivity Analysis

Four distinct study groups were redefined by the lower normal limit of albumin (3.5 g/dL) and the median value of globulin (2.7 g/dL) as follows: group A (*n* = 103), albumin ≥ 3.5 g/dL and globulin < 2.8 g/dL; group B (*n* = 133), albumin ≥ 3.5 g/dL and globulin ≥ 2.8 g/dL; group C (*n* = 154), albumin < 3.5 g/dL and globulin < 2.8 g/dL; and group D (*n* = 164), albumin < 3.5 g/dL and globulin ≥ 2.8 g/dL. Additional sensitivity analyses resulted in similar findings in the unadjusted and fully adjusted models (Table 4). Similarly, group D was associated with the highest adjusted risk of all-cause and CV mortality (HR (95% CI), 4.02 (1.82–8.91) and 5.14 (1.74–15.16), respectively). The trend of the increasing risk of overall and CV mortality remained from group A to group D (both *p*-values for the trend < 0.005).

## 4. Discussion

In this study cohort, we evaluated the interaction between serum albumin and globulin and found that albumin and globulin had a synergistic effect in predicting the risk of death in incident PD patients. Patients with high albumin and low globulin had the lowest risk of all-cause and CV mortality, while those with low albumin and high globulin carried the highest risk. Multivariate adjustment analyses further confirmed that this association was independent of many clinical crucial confounders. These associations still existed in the sensitivity analyses using different cut-off points for albumin, thus strengthening the robustness of our findings.

Albumin is the most abundant serum protein, which is synthesized in the liver and functions to maintain normal microvascular permeability and the PH blood balance and acts to scavenge free radicals as an antioxidant [13]. Albumin can be regarded in not only the nutritional but also the inflammatory status. Poor nutrition intake is one of the common causes of low albumin in the general population. In CKD, low serum albumin can be caused by a reduced nutrition intake, altered catabolism, reduced anabolism, chronic inflammation and dialysis treatment [11]. Malnutrition should be distinguished from protein energy wasting (PEW). Malnutrition usually refers to insufficient calorie intake or the inability to meet one’s metabolic needs due to dietary restrictions or anorexia. PEW is a peculiar entity that describes the coexistence of hypoalbuminemia, inflammation and muscle wasting despite sufficient nutrient intake in CKD, particularly ESRD [7,14,15]. Albumin is one of the most used laboratory measurements to define PEW. In the Modification of Diet in Renal Disease (MDRD) study, low albumin independently predicted all-cause mortality in stage 3 and 4 CKD patients over a 10-year follow-up [16]. Moreover, higher albumin concentrations were related to a lower risk of higher aortic pulse wave velocity and, therefore, lower arterial stiffness [17]. Consistent with previous studies in PD patients [18,19], we also found a low albumin level (group C and group D) associated with a high mortality risk. The prevalent PEW is an important risk factor for morbidity and mortality in patients on dialysis, contributing mainly to the higher risk of mortality in the low albumin groups in our study.

The BMI is widely used as a valid nutritional indicator to stratify under- or overnutrition in the general population. However, the BMI distribution did not differ among our study groups based on the serum albumin and globulin levels, although albumin is a nutritional marker. One of the reasons could be the susceptibility of CKD patients to fluid retention, affecting the BMI measurements. Additionally, both high muscle mass and excessive body fat can lead to a high BMI, but the BMI cannot distinguish muscle from fat mass [20]. Furthermore, the BMI also cannot distinguish subcutaneous fat from visceral fat, and visceral fat was more related to oxidative stress and inflammation [21,22]. Therefore, the BMI may not be an accurate biomarker for the evaluation of nutrition in dialysis patients.

Globulin is the major constituent of non-albumin serum protein and is mainly composed of immunoglobulin, which can carry sex hormones and fatty acids, and is involved in immunologic and inflammatory processes [23,24]. Therefore, the globulin level can serve as a marker of a chronic inflammatory response, and higher globulin levels indicate a more severe inflammation. Higher CRP was independently associated with a higher prevalence of CV disease in stage 3 and 4 CKD patients [25]. Recent studies have reported that inflammatory cytokines and CRP are powerful predictors of mortality in PD patients [26,27]. Globulin was also reported to be associated with a poor prognosis in patients with different types of cancer, including cervical cancer, rectal cancer, gastric cancer and nasopharyngeal carcinoma [12,28,29,30]. Similarly, we also found a high globulin level (group B and group D) associated with a high mortality risk in PD. The observed predictive role of high globulin cannot be merely explained by low albumin because of the significant survival difference between groups A and B, both of which did not have hypoalbuminemia. Chronic low-grade inflammation in CKD was attributed to oxidative stress produced by the uremic milieu; amplified production and a reduced clearance of proinflammatory mediators, metabolic acidosis and intestinal dysbiosis, which facilitates the translocation of the gut bacteria and provokes an inflammatory response [31]. Furthermore, a deficiency in vitamin D can cause dysregulation of the immune system, and disturbances in the endocrine function of adipose tissue can lead to the homeostatic deregulation of inflammatory cytokines [32]. In PD patients, some unique factors contributing to chronic inflammation include endotoxin exposure by conventional glucose-based bioincompatible dialysate and PD catheter-related infection, such as peritonitis, exit site infection and tunnel tract infection. New targeted therapies can only be developed with a deep understanding of the complex mechanisms behind inflammation in CKD in order to effectively mitigate inflammation and improve patient survival.

There are several studies addressing the predictive role of albumin and globulin in the mortality risk in CKD patients, and they use the albumin–globulin ratio as the predictor. For example, a Taiwanese retrospective study of stages 3–5 CKD patients by Wu et al. reported that the albumin–globulin ratio was a statistically significant predictor of the overall and CV mortality after adjusting for clinically important confounders [33]. Later, consistent results were shown by a study of Chinese CKD patients that enrolled over 3000 participants over a median follow-up of 10 years [34]. A low albumin–globulin ratio can be caused by low albumin or high globulin, so it is unclear which one is associated with poor survival and whether the coexistence of them may lead to a worse survival. Our novel finding was the association of the coexistence of low albumin and high globulin with the highest risk of overall and CV mortality. Taking group A as the reference, the mortality risk of group D was higher than that of group B and group C, both of which only had either low albumin or high globulin. The HR of mortality for group D was higher than those for group B and C, indicating the synergistic effect of low albumin and high globulin on mortality prediction. Hypoalbuminemia and inflammation are interrelated and contributed to the development of atherosclerosis, which can cause cardiovascular disease [35], the main cause of death in our dialysis patients. The presence of one or more of the three components (malnutrition, inflammation and background atherosclerosis) can be diagnosed as malnutrition–inflammation–atherosclerosis (MIA) syndrome, which is significantly linked to mortality in dialysis patients [36]. A Japanese study of 5813 patients found that the 36-month all-cause mortality rate was directly correlated with the number of MIA factors [37]. This can partly explain the mechanism underlying our notion that the combination of low albumin and high globulin was related to a worse survival than either of them alone.

This study should also be interpreted with several potential limitations. First, the residual confounding factors could not be completely excluded for the retrospective observational study, despite our efforts to account for the many relevant clinical parameters, such as age, comorbidities, pharmacotherapies, biochemical data and PD-associated parameters. Second, an inherent limitation was that association is not equivalent to causation for the retrospective study which was subjected to various biases and reverse causation due to the unmeasured confounders. Risk factors can be determined through epidemiological studies, while causal factors can only be determined through randomized controlled trials. The albumin–globulin interaction may only be a valuable biomarker that reflects and integrates various important causal factors and their synergistic effects. Third, we assessed albumin and globulin only once at the baseline. The changes in albumin and globulin over time require further study. Fourth, there is no uniform reasonable cut-off values for albumin and globulin for their interaction, and we first adopted the median values of both parameters for the analyses. However, sensitivity tests using different albumin cut-off values confirmed the powerful predictive capacity of the albumin–globulin interactions. Finally, given the single-center nature of the study design, these findings may not be able to be extrapolated to other ethnic populations. Nonetheless, to our knowledge, this is the first study to investigate the combination of albumin and globulin with mortality in PD patients.

In conclusion, our study observed the synergistic effect of albumin and globulin on the mortality risk in patients undergoing PD, indicating that those with either low albumin or high globulin had a higher mortality risk, especially for those with both low albumin and high globulin. This relationship was independent of many clinical confounders and remained consistent in the sensitivity analyses. Our research results support the notion that clinicians should pay more medical attention to PD patients with low albumin or high globulin, especially those with both of them, while providing medical care. In view of the lack of convincing targeted therapies, the screening and early detection of malnutrition and inflammation and immediate implementation of some effective interventions are essential to improve the prognosis of PD patients. Further studies are needed to explore the mechanisms underlying this phenomenon and how to improve the clinical outcomes in those high-risk patients.

## Figures and Tables

**Table 1:** Baseline characteristics of 554 patients stratified by the median values of albumin and globulin.

Variable	Group A *n* = 135	Group B *n* = 165	Group C *n* = 122	Group D *n* = 132	*p*-Value
	ALB ≥ 3.3 g/dL GLB < 2.8 g/dL	ALB ≥ 3.3 g/dL GLB ≥ 2.8 g/dL	ALB < 3.3 g/dL GLB < 2.8 g/dL	ALB < 3.3 g/dL GLB ≥ 2.8 g/dL	
**Sex**					0.141
Male	67 (49.6%)	70 (42.4%)	68 (55.7%)	60 (45.5%)	
Age (years)	47.03 ± 13.25	53.34 ± 15.74	52.83 ± 15.82	58.48 ± 15.2	<0.001 *
BMI (kg/m^2^)	23.43 ± 3.73	23.32 ± 3.92	23.65 ± 4.12	23.41 ± 4.01	0.918
**Comorbidity**					
Hypertension	117 (86.7%)	130 (78.8%)	111 (91%)	113 (85.6%)	0.033 *
Diabetes mellitus	22 (16.3%)	43 (26.1%)	54 (44.3%)	67 (50.8%)	<0.001 *
Cardiovascular disease	26 (19.3%)	47 (28.5%)	58 (47.5%)	69 (52.3%)	0.003 *
**Medication prescription**					
ACE inhibitor/ARB	87 (64.4%)	84 (50.9%)	87 (71.3%)	85 (64.4%)	0.003 *
**Laboratory data**					
Calcium (mg/dL)	8.42 ± 0.75	8.42 ± 0.78	8.25 ± 0.7	8.32 ± 0.71	0.173
Cholesterol (mg/dL)	182.56 ± 40.99	182.6 ± 45.11	194.33 ± 57.35	196.16 ± 58.33	0.049 *
Creatinine (mg/dL)	11.28 ± 3.35	10.08 ± 2.87	9.88 ± 3.28	8.69 ± 2.86	0.273
Ferritin (ng/mL)	325.25 ± 318.25	376.42 ± 517.14	304.86 ± 307.18	484.11 ± 659.26	<0.001 *
GPT (U/L)	20.93 ± 17.94	24.23 ± 42.47	19.73 ± 15.28	21.29 ± 14.6	0.034 *
Hemoglobin (g/dL)	8.36 ± 1.48	8.67 ± 1.26	8.64 ± 1.22	8.77 ± 1.16	0.040 *
i-PTH (pg/mL)	473.43 ± 357.23	431.45 ± 339.41	358.57 ± 244.13	361.59 ± 316.89	0.012 *
Phosphate (mg/dL)	5.89 ± 1.28	5.4 ± 1.24	5.44 ± 1.3	5.29 ± 1.36	0.452
Triglyceride (mg/dL)	120.86 ± 58.58	145.41 ± 98.97	138.56 ± 86.27	152.49 ± 136.4	0.080 *
WBC count (/μL)	6.55 ± 2.07	7.5 ± 2.44	7.55 ± 2.59	8.35 ± 2.53	0.142
**Peritoneal Dialysis related parameters**					
D/P creatinine at 4 h	0.66 ± 0.13	0.66 ± 0.11	0.7 ± 0.12	0.69 ± 0.14	0.007 *
Weekly total Kt/V urea	2.15 ± 0.45	2.11 ± 0.48	1.97 ± 0.63	1.94 ± 0.42	0.001 *
nPNA (g/kg/day)	1.15 ± 0.29	1.04 ± 0.26	1.01 ± 0.29	1.01 ± 0.36	0.001 *
Residual renal function (mL/min/1.73 m^2^)	3.21 ± 1.51	3.19 ± 1.61	2.86 ± 1.95	2.85 ± 1.57	0.135

Values are expressed as the mean ± standard deviation, median and interquartile range or number (percentage). Abbreviations: ACE inhibitor, angiotensin-converting enzyme inhibitor; ARB, angiotensin II receptor blocker; GPT, glutamic–pyruvic transaminase; WBC, white blood cell; PTH, parathyroid hormone; D/P creatinine, dialysate-to-plasma creatinine ratio; and nPNA, normalized protein nitrogen appearance. * *p*-value < 0.05.

**Table 2:** Associations of the albumin–globulin groups with study outcomes by Cox models.

Group	All-Cause Mortality				Cardiovascular Mortality			
	Crude HR (95% CI)	*p*-Value	Adjusted HR (95% CI)	*p*-Value	Crude HR (95% CI)	*p*-Value	Adjusted HR (95% CI)	*p*-Value
A: ALB ≥ 3.3 g/dL and GLB < 2.8 g/dL	1.00	-	1.00	-	1.00	-	1.00	-
B: ALB ≥ 3.3 g/dL and GLB ≥ 2.8 g/dL	3.54 (1.74,7.22)	0.001 *	2.74 (1.33,5.65)	0.006 *	2.99 (1.4,6.38)	0.005 *	2.67 (1.05,6.82)	0.04 *
C: ALB < 3.3 g/dL and GLB < 2.8 g/dL	4.13 (1.93,8.83)	<0.001 *	2.22 (1.03,4.82)	0.043 *	3.67 (1.67,8.04)	0.001 *	2.56 (0.96,6.87)	0.061
D: ALB < 3.3 g/dL and GLB ≥ 2.8 g/dL	8.94 (4.32,18.51)	<0.001 *	3.87 (1.83,8.20)	<0.001 *	8.20 (3.93,17.12)	<0.001 *	5.65 (2.23,14.34)	<0.001 *
*p*-value for trend	<0.001 *		0.001 *		<0.001*		<0.001 *	

Abbreviations: HR, hazard ratio; ALB, albumin; and GLB, globulin. * *p*-value < 0.05.

**Table 3:** Logistic regression model-based estimated odds ratios of the low albumin and high globulin group (group D) compared with other albumin–globulin groups (reference).

Variables	Crude OR (95% CI)	*p*-Value	Adjusted OR (95% CI)	*p*-Value
Sex	0.88 (0.60,1.31)	0.531	-	-
Age (years)	1.03 (1.02,1.05)	<0.001 *	-	-
BMI (kg/m^2^)	0.99 (0.95,1.05)	0.913	-	-
**Comorbidity**				
Hypertension	1.06 (0.61,1.85)	0.828	-	-
Diabetes mellitus	2.62 (1.76,3.92)	<0.001 *	2.00 (1.22,3.27)	0.006 *
Cardiovascular disease	2.43 (1.63,3.63)	<0.001 *	1.66 (1.01,2.74)	0.046 *
**Medication prescription**				
ACE inhibitor/ARB	1.15 (0.77,1.73)	0.501	-	-
**Laboratory data**				
Calcium	0.91 (0.70,1.19)	0.497	-	-
Cholesterol	1.00 (1.00,1.01)	0.061	-	-
Creatinine	0.81 (0.75,0.88)	<0.001 *	0.86 (0.79,0.95)	0.001 *
Ferritin	1.00 (1.00,1.001)	0.008 *	-	-
GPT	0.99 (0.99,1.01)	0.828	-	-
Hemoglobin	1.13 (0.97,1.33)	0.105	-	-
i-PTH	0.99 (0.99,1.00)	0.069	-	-
Phosphate	0.84 (0.72,0.99)	0.035 *	-	-
Triglyceride	1.00 (1.00,1.003)	0.129	-	-
WBC count	1.18 (1.10,1.29)	<0.001 *	1.14 (1.04,1.25)	0.005 *
**Peritoneal dialysis related** **parameters**				
D/P creatinine at 4 h	3.54 (0.68,18.63)	0.134	-	-
Weekly total Kt/V urea	0.54 (0.35,0.84)	0.006 *	-	-
nPNA	0.51 (0.25,1.06)	0.071	-	-
Residual renal function	0.90 (0.80,1.03)	0.142	-	-

Abbreviations: ACE inhibitor, angiotensin-converting enzyme inhibitor; ARB, angiotensin II receptor blocker; GPT, glutamic–pyruvic transaminase; WBC, white blood cell; PTH, parathyroid hormone; D/P creatinine, dialysate-to-plasma creatinine ratio; and nPNA, normalized protein nitrogen appearance. * *p*-value < 0.05.

**Table 4:** Sensitivity analyses.

Group	All-Cause Mortality				Cardiovascular Mortality			
	Crude HR (95% CI)	*p*-Value	Adjusted HR (95% CI)	*p*-value	Crude HR (95% CI)	*p*-Value	Adjusted HR (95% CI)	*p*-Value
A: ALB ≥ 3.5 g/dL and GLB < 2.8 g/dL	1.00	-	1.00	-	1.00	-	1.00	-
B: ALB ≥ 3.5 g/dL and GLB ≥ 2.8 g/dL	2.907 (1.42,5.95)	0.004 *	2.21 (0.98,4.95)	0.055	2.844 (1.13,7.17)	0.027 *	3.118 (1.02,9.51)	0.046 *
C: ALB < 3.5 g/dL and GLB < 2.8 g/dL	3.418 (1.69,6.93)	0.001 *	2.07 (0.91,4.74)	0.084	3.778 (1.54,9.27)	0.004 *	2.533 (0.83,7.74)	0.103
D: ALB < 3.5 g/dL and GLB ≥ 2.8 g/dL	9.85 (5.03,19.29)	<0.001 *	4.02 (1.82,8.91)	0.001^*^	9.584 (4.05,22.69)	<0.001 *	5.141 (1.74,15.16)	0.003 *
*p*-value for trend	<0.001 *		<0.001 *		<0.001 *		0.002 *	

Abbreviations: HR, hazard ratio; ALB, albumin; and GLB, globulin. The whole cohort was divided by the lower limit of normal for albumin and the median value for globulin. * *p*-value < 0.05.

## Data Availability

The datasets generated or analyzed during the current study are available from the corresponding author upon reasonable request.
